# Case report of intrafamilial variability in autosomal recessive centronuclear myopathy associated to a novel *BIN1 *stop mutation

**DOI:** 10.1186/1750-1172-5-35

**Published:** 2010-12-03

**Authors:** Johann Böhm, Uluç Yiş, Ragıp Ortaç, Handan Çakmakçı, Semra Hız Kurul, Eray Dirik, Jocelyn Laporte

**Affiliations:** 1 Department of Neurobiology and Genetics, IGBMC (Institut de Génétique et de Biologie Moléculaire et Cellulaire), Illkirch, France; 2 Inserm, U964, Illkirch, France; 3 CNRS, UMR7104, Illkirch, France; 4 Université de Strasbourg, Strasbourg, France; 5 Collège de France, chaire de génétique humaine, Illkirch, France; 6 Division of Child Neurology, Gaziantep Children's Hospital, Gaziantep, Turkey; 7 Department of Pathology, Behçet Uz Training Hospital for Children, İzmir, Turkey; 8 Department of Radiology, Dokuz Eylül University School of Medicine, İzmir, Turkey; 9 Department of Pediatrics, Dokuz Eylül University School of Medicine, İzmir, Turkey

## Abstract

Centronuclear myopathies (CNM) describe a group of rare muscle diseases typically presenting an abnormal positioning of nuclei in muscle fibers. To date, three genes are known to be associated to a classical CNM phenotype. The X-linked neonatal form (XLCNM) is due to mutations in *MTM1 *and involves a severe and generalized muscle weakness at birth. The autosomal dominant form results from *DNM2 *mutations and has been described with early childhood and adult onset (ADCNM). Autosomal recessive centronuclear myopathy (ARCNM) is less characterized and has recently been associated to mutations in *BIN1*, encoding amphiphysin 2. Here we present the first clinical description of intrafamilal variability in two first-degree cousins with a novel *BIN1 *stop mutation. In addition to skeletal muscle defects, both patients have mild mental retardation and the more severely affected male also displays abnormal ventilation and cardiac arrhythmia, thus expanding the phenotypic spectrum of *BIN1*-related CNM to non skeletal muscle defects. We provide an up-to-date review of all previous cases with ARCNM and *BIN1 *mutations.

## Background

Centronuclear myopathies (CNM) are a group of congenital disorders characterized by hypotonia and skeletal muscle biopsies typically showing small rounded fibers with central nuclei [1-4]. Abnormal nuclear positioning is seen in several myopathies, but clinical, genetic and pathological factors clearly distinguish these myopathies from CNM. Three CNM classes have been described: the severe neonatal X-linked form, also called myotubular myopathy (XLCNM, OMIM 310400), the autosomal recessive form with childhood onset (ARCNM, OMIM 255200), and the autosomal dominant form with adult onset (ADCNM, OMIM 160150). Myotubularin (*MTM1*) is mutated in XLCNM [5] and belongs to a large family of ubiquitously expressed phosphoinositide phosphatases implicated in intracellular vesicle trafficking [6-8]. The large GTPase dynamin 2 (*DNM2*), mutated in ADCNM, is a mechanochemical enzyme and a key factor in membrane trafficking and endocytosis [9-11]. Amphiphysin 2 (*BIN1*) is mutated in ARCNM and possesses an N-terminal BAR domain able to sense and bend membranes and a SH3 domain mediating protein-protein interactions [12,13]. A muscle-specific isoform is implicated in T-tubule biogenesis and contains a polybasic residue sequence binding to phosphoinositides [14]. Only 4 unrelated individuals with *BIN1 *mutations have been molecularly and clinically characterized to date [12,15] and this report is the first description of intrafamilal variability in two patients from a consanguineous family. Clinical analysis of respiratory and cardiac involvement diagnosed for the more severely affected male patient expand the phenotypic spectrum in autosomal recessive centronuclear myopathy. It is furthermore the first time that patients with a *BIN1 *mutation are analyzed by whole-body MRI and the results contrast previous findings on *DNM2*-related CNM.

## Clinical report and results

**Figure 1:**
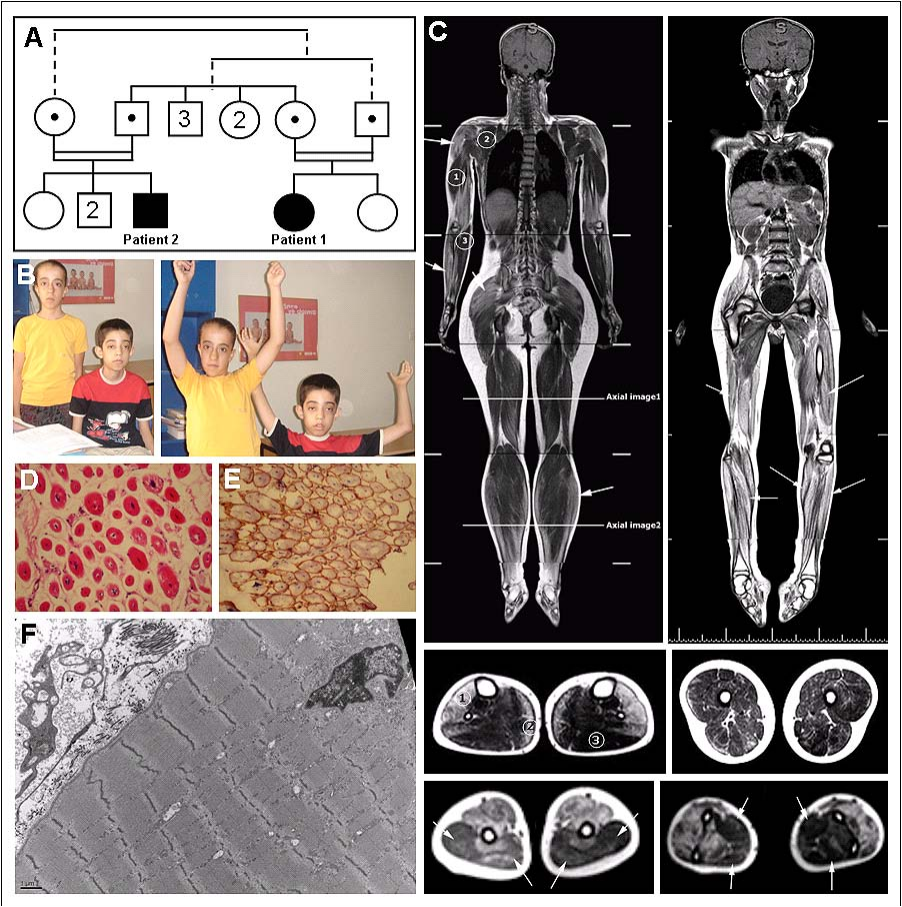
**Clinical, histological and MRI features in the patients**. (A) Pedigree of the consanguineous family of both ARCNM patients. Segregation of the mutation (c.1717C > T in NM_139343) in the tested individuals is depicted as black dots; both patients are homozygous for the mutation while all four parents are heterozygous. Other individuals were not tested. The related parents have 3 brothers and 2 sisters, and the male patient has 1 sister and 2 brothers. (B) Photos of both patient 1 (on the left) and patient 2 (right). (C) T1 weighted coronal whole body MRI images of patients 1 (left) and 2 (right) and axial displays of the femoral and the lower leg regions (below, middle panel) demonstrate prominent fatty involvement of soleus [2], tibialis anterior, peroneal and extensor muscles [1], but sparing of the gastrocnemius [3]. All thigh muscle groups were affected without selective pattern. Imaging of upper limb demonstrated relative sparing of triceps [1] and arrows, left picture, lower panel], subscapularis [2] and flexor [3] and arrows, right picture, lower panel] muscle groups. (D) Muscle biopsy from patient 2 showing small rounded fibers with a high percentage of central nuclei, variable fiber size, extensive fibrosis, and (F) electron microscopy image showing myofibrillar disorganization. (E) Muscle biopsy of patient 2 with normal Dystrophin labeling.

Patient 1 is a 13 year old girl belonging to a consanguineous family from Turkey without ancestral history of neuromuscular disorders (Figure 1A-B). There were no complications during pregnancy, antenatal signs for muscle disorders as polyhydramnios and reduced fetal movements were not noted. Hypotonia was diagnosed at birth and motor development was delayed: head control was achieved at 6 months, walking at 18 months and running at 36 months. Muscle weakness was predominantly proximal, accompanied by mild facial weakness, ptosis and ophtalmoplegia/paresis. Tendinous reflexes were absent and she has no contractures. Although she has mild mental retardation (IQ 60), speech development was normal and she integrated the regular educational system. Echocardiography, electrocardiography and electroneuromyography were normal, and there were no indications of myotonia or neuromuscular junction abnormalities. Serum creatine kinase was mildly elevated [380 IU/L (70-150); normal range 60 - 320 IU/L]. She is currently walking independently but she has difficulty climbing stairs and running. Pulmonary function tests are normal. Patient 1 has one non-affected sister and none of the parents displays clinical features of a muscle disorder.

Patient 2, a 14 year old boy, is the first-degree cousin of patient 1 and belongs to a second consanguineous family loop (Figure 1A-B). The course of the disease was rather similar to patient 1 with normal pregnancy, hypotonia at birth, delayed motor milestones and normal speech development despite a mild mental retardation (IQ 60). Head control was achieved at 6 months, walking at 18 months and running at 36 months. Likewise, patient 2 presents a predominantly proximal muscle weakness, absent tendinous reflexes, facial weakness, ptosis and opthalmoplegia/paresis. However, his phenotype is more severe as he is not able to walk independently since the age of 10 years and is wheelchair-bound. Furthermore, the degree of ophtalmoplegia/paresis and ptosis is more prominent than in patient 1. In addition, electrocardiography and HOLTER examination revealed premature ventricular complexes while echocardiography was normal. Serum creatine kinase was 450 IU/L (70-150) and electromyography revealed myopathic changes in all muscle groups. He needs non-invasive respiratory support for four hours per day. Patient 2 has healthy parents and 3 non-affected siblings.

Whole body MRI of both patients revealed similar results with increased signals on T2 and T1 weighted images in thigh muscles, upper and lower extremities which are consistent with fatty infiltrations. Detailed axial imaging of the femoral and crural regions of patient 1 revealed prominent fatty involvement of soleus, tibialis anterior, peroneal and extensor muscles, but sparing of the gastrocnemius (Figure 1C). All thigh muscle groups were affected without selective pattern. Imaging of upper limb demonstrated relative sparing of triceps, subscapularis and flexor muscle groups (Figure 1C). No abnormalities of brain, heart or other organs were noted.

Diagnosis of CNM for both patients was suggested on muscle biopsies showing numerous centrally located and partially clustered nuclei, variable fiber size, type 1 fiber predominance, extensive myofibrillar disorganization (Figure 1F) and fibrosis (Figure 1D). Dystrophin expression was normal (Figure 1E). *BIN1 *sequencing revealed a homozygous nonsense mutation in exon 20 in both patients (c.1717C > T; p.Gln573stop). Both patients have healthy parents heterozygous for this mutation.

## Discussion

By direct sequencing of the 20 *BIN1 *exons and the adjacent splice-relevant regions we identified the novel homozygous nonsense *BIN1 *mutation p.Gln573stop in two first-degree cousins from a consanguineous family. Both patients present predominantly proximal muscle weakness and classical features of CNM with a general progressive hypotonia involving facial weakness and ptosis. Ophtalmoplegia/paresis, as seen in both patients, is not a common sign of ARCNM (Table 1), while it is consistently reported for the X-linked form. However, Ophtalmoplegia/paresis often evolves over time and might not have been diagnosed in all *BIN1 *patients due to their young age. Whole body MRI showed fatty infiltrations of different muscle groups with selective muscle involvement in the lower leg, and a general muscle involvement in the thigh. This contrasts MRI findings in *DNM2*-related centronuclear myopathies where prominent fatty atrophy was predominantly documented in the lower leg muscles, but only in specific thigh muscles: increased signals were reported for adductor longus, semimembranosus, rectus femoris, biceps femoris, and vastus intermedius muscles, while the adductor magnus, gracilis, sartorius, semitendinosus, vastus lateralis, and vastus medialis muscles were only minimally affected [16].

**Table 1:** Clinical comparison of all patients with known BIN1 mutations

	Patient	Sex	Mutation^1^	AA change	Origin	Age of onset	Age^2^	Central nuclei	Pregnancy	Ventilation	Muscle weakness	Facial weakness	Ptosis	Ophtalmoplegia/ paresis	Other phenotypes	Cognitive development	Cardiac function	Reference
Family 1	AAT68	male	c.105G > T	p.LysK35Asn	India	birth	12	++	reduced fetal movements, oligohydramnios, IUGR^3^	normal	proximal, slowly progressive	no	yes	yes	contractures at birth	normal	normal	[12]
	
	ACC82	female	c.105G > T	p.LysK35Asn	India	birth	died at 1year	+	reduced fetal movements, oligohydramnios, IUGR	normal	proximal	no	no	no	contractures at birth	hypodevelopment of frontal lobes	died from myocarditis	[12]
	
	ADS5	female	no DNA available	n.d.	India	birth	died at 18 hrs	n.d.	reduced fetal movements, oligohydramnios, IUGR, premature birth	lung hypoplasia, ventilated from birth, died from respiratory failure	no spontaneous movements	n.d.	n.d.	n.d.	severe joint contractures at birth	n.d.	prenatal heart enlarged, postnatal ECG normal	[12]

Family 2	ADR71	male	c.451G > A	p.Asp151Asn	Iraq	8	35	++	normal	n.d.	proximal	n.d	no	no	no	normal	normal	[12]

Single case	AEY47	male	c.461G > A	pArg154Gln	Morocco	11	21	++	normal	respiratory insufficiency	diffuse atrophy, slowly progressive	yes	yes	yes	Scapular winging, hyperlordosis, left-sided kyphoscoliosis	IQ 70	normal	[15]

This family	AFG89 (patient 1)	female	c.1717C > T	p.Gln573stop	Turkey	birth	13	++	normal	normal	proximal	yes	yes	yes	no	IQ 60	normal	this study
	
	AFG92 (patient 2)	male	c.1717C > T	p.Gln573stop	Turkey	birth	14	++	normal	respiratory insufficiency	proximal	yes	yes	yes	no	IQ 60	cardiac arrhythmia	this study

Family 3	LF41	male	c.1723A > T	p.Lys575stop	Iraq	birth	14	++	normal	respiratory insufficiency	proximal, slowly progressive	yes	yes	yes	scoliosis	normal	normal	[12,18]

^1^Nucleotide numbering from the A of the ATG start codon in *BIN1-iso1 *reference sequence (NM_139343).

^2^At time of publication (yrs)

^3^Intrauterine growth restriction

^4^Mejaddam et al., 2009 reported the detailed histopathological characterization of patient LF41 described in Nicot et al., 2007.

This is consistent with the observation that ARCNM patients with *BIN1 *mutations predominantly display a proximal muscle weakness (Table 1), whereas ADCNM patients with *DNM2 *mutations rather present involvement of the distal muscles [17]. Characterization of additional ARCNM and ADCNM patients is required to confirm that MRI could be used as a differential marker to direct genetic diagnosis.

To our knowledge, this is the second documented ARCNM family with more than one molecularly characterized member. Nicot et al. reported a family with three affected members, two of which died within the first year of life, precluding a long term comparison of clinical signs [[12], Table 1]. In the present study we observed a clear intra-familiar variability, which might be linked to gender or modifier genes differing between individuals. Patient 2 does not walk independently, has a more pronounced ophtalmoplegia/paresis and ptosis, and electroneuromyography revealed myopathic changes not detected in patient 1. Patient 2 has also been diagnosed for additional respiratory system and cardiac involvements. Abnormal ventilation has been documented for two other autosomal recessive cases [[15,18], Table 1] and a further patient died at birth due to respiratory failure [[12], Table 1]. Cardiac arrhythmia was stated for patient 2, while ECG examinations did not reveal abnormalities for patient 1. As cardiac abnormalities have been reported for another ARCNM patient who died from myocarditis shortly after birth [[12], Table 1], we suggest careful cardiac function examinations and long term follow-up of patients with *BIN1 *mutations.

A mild mental retardation, as seen in both patients, has recently been described in another ARCNM patient [[15], Table 1]. Mental impairment was not noticed in the other *BIN1*-patients and is rarely present in other CNM forms. However, decreased synaptic vesicle recycling in the murine brain was described in amphiphysin 1 knockout mice, suggesting a possible pathological mechanism affecting cognitive abilities [19]. We though cannot exclude that the mental retardation might not be correlated to the *BIN1 *mutation, especially in a consanguineous family.

BIN1 was initially identified as a c-Myc interacting pro-apoptotic tumor suppressor [20]; *BIN1 *expression is reduced in several cancers and mice deficient for BIN1 develop more aggressive tumors [21,22]. However, no tumors were reported in the small set of ARCNM patients with *BIN1 *mutations.

The novel p.Gln573stop mutation described in this study is in direct spatial proximity to the previously identified p.Lys575stop mutation, which results in the expression of a truncated protein with decreased dynamin 2 binding [12]. At the time of publication, the p.Lys575stop patient was 17 years old, able to walk short distances, had normal cognitive development and no cardiac involvement [[12,18], Table 1], contrasting the present study. Table 1 gives an overview of the clinical manifestations of all currently published *BIN1 *patients. Disease onset at birth was stated for all patients except for ADR71 and AEY47 (p.Asp151Asn and pArg154Gln, respectively), harboring adjacent missense mutations in the BAR-domain and presenting a generally milder etiopathology. The identification of more *BIN1 *mutations and respective detailed clinical descriptions might help to establish an unambiguous genotype/phenotype correlation and to clarify if the most 3' *BIN1 *exon represents a hot spot prone to mutations.

In conclusion, this study expands the phenotypic spectrum of *BIN1*-related centronuclear myopathy and is the first clinical description of intrafamilial variability in a consanguineous CNM family.

## Competing interests

The authors declare that they have no competing interests.

## Authors' contributions

JB carried out the molecular genetics studies. UY, SHK and ED carried out the clinical investigation. RO carried out the histologic studies. HC carried out MRI. JB and JL wrote the manuscript. JL conceived and coordinated the study. All authors have read and approved the final manuscript

## Consent

Written informed consent was obtained from the patient's parents for publication of these case reports and accompanying images.
